# Editorial for the Special Issue “Gut Microbiome in Homeostasis and Disease, 2nd Edition”

**DOI:** 10.3390/microorganisms13102297

**Published:** 2025-10-03

**Authors:** Michael Doulberis

**Affiliations:** 1 Department of Gastroenterology and Hepatology, Department of Medicine, Zurich University Hospital, 8091 Zurich, Switzerland; michael.doulberis@usz.ch; 2 Division of Gastroenterology and Hepatology, Medical University Department, Kantonsspital Aarau, 5001 Aarau, Switzerland

The gut microbiome has emerged lately as a cornerstone of human (patho)physiology, intricately involved in the complex regulation of the immune system, metabolic pathways, and neurobehavioral patterns. Although the composition and activity of the gut microbial ecosystem are central to maintaining homeostasis, its disruption—commonly defined as dysbiosis—has been increasingly implicated in a wide spectrum of pathologies, from localized gastrointestinal disorders to systemic metabolic, autoimmune, and neuropsychiatric diseases [1,2].

Over the past decades, research has shifted from descriptive studies characterizing merely microbial communities towards more sophisticated investigations that integrate multi-omics, mechanistical insights, and clinical applications. Nevertheless, fundamental questions remain unanswered: How are we able to distinguish between association and etiology, identify microbial signatures that are robust across populations, and translate promising experimental findings into effective therapies in clinical real-life [3,4]?

This Special Issue of *Microorganisms*, entitled “Gut Microbiome in Homeostasis and Disease, 2nd Edition”, is online and free available at the following link: https://www.mdpi.com/journal/microorganisms/special_issues/W786Z6GXY9, accessed on 29 September 2025.

It encompasses thirteen articles (ten original and three reviews) that collectively address aforementioned unanswered questions. The contributions (numbered 1–13 according to publication date online) cover a broad spectrum—from infections such as lymph node tuberculosis (LNTB), to metabolic diseases such as metabolic (dysfunction) associated steatotic liver disease (MASLD) and cardiovascular disorders, and to inflammatory bowel disease (IBD), autoimmune conditions, neurodevelopmental syndromes, and innovative therapeutic approaches including fecal microbiota transplantation (FMT) and probiotic interventions. Systematic reviews further provide clarity on controversial taxa such as *Blautia*, whereas conceptual perspectives stress the microbiome’s striking contribution in diseases such as rheumatoid arthritis.

Taken together, the articles not only highlight the remarkable breadth of microbiome research but also emphasize the translational potential of targeting the gut microbiota for disease prevention and therapy. In the following sections, the individual contributions will be summarized, common themes will be highlighted, and further implications will be discussed for future directions in this research field.

Long et al. (contributor 1) investigated patients with LNTB using a combined metagenomic and metabolomic approach. Their findings revealed a remarkable reduction in bacterial taxa central to short-chain fatty acid (SCFA) production, including *Ruminococcus*, *Faecalibacterium*, *Roseburia*, and *Blautia*. This effect was accompanied by alterations in plasma metabolites linked to inflammation and energy metabolism. The integrated analysis suggested that depletion of SCFA-producing bacteria may contribute to impaired host defense and disease progression, thereby shedding light on novel therapeutic strategies targeting microbial metabolism in tuberculosis.

A broader perspective on metabolic health is provided by the systematic review of Chanda et al. (contributor 13), which examined the ambiguous role of the genus *Blautia* in obesity. Although certain studies connect *Blautia* to beneficial metabolic effects and probiotic potential, others link its expansion with weight gain and metabolic dysfunction. The review deduces that the current evidence remains inconclusive, particularly at the genus level, and that species-specific effects are likely to drive the observed discrepancies. This work highlights the significance of moving beyond taxonomic resolution toward functional profiling, to better elucidate how microbial contributions shape host metabolic phenotypes.

In the metabolic realm, Munte et al. (contributor 2) focused on MASLD, elucidating the role of *Faecalibacterium prausnitzii*, a prototypical butyrate-producing bacterium. Their analysis exhibited that the abundance of *F. prausnitzii* was markedly reduced in MASLD patients, particularly in those with advanced liver fibrosis. Although sodium-butyrate supplementation enhanced fatty liver in murine models, direct gavage of live butyrate-producing strains, including *F. prausnitzii* and *Coprococcus* comes, failed to ameliorate disease outcomes. These findings suggest that therapeutic benefit may depend more on microbial functional outputs rather than solely on the presence of concrete bacterial strains, emphasizing the complexity of host–microbe crosstalk in metabolic liver disease.

Furthermore, Siu et al. (contributor 8) investigated the effects of a novel probiotic formulation on gastrointestinal comorbidities in individuals with psoriasis, a condition increasingly linked to so-called “gut–skin axis” disturbances. Over an eight-week intervention, responders to probiotic treatment exhibited improvements in stool form and quality-of-life indices, accompanied by shifts in gut microbial composition—namely an enrichment of *Lactobacillus acidophilus* and *Parabacteroides distasonis* and a reduction in Oscillibacter and *Bacteroides vulgatus*. Such findings suggest that probiotic interventions may not just milden gastrointestinal symptoms, but even enhance systemic manifestations in chronic inflammatory diseases, therefore further highlighting the therapeutic potential of microbiome modulation.

In the context of inflammatory bowel disease (IBD), Zobrist et al. (contributor 4) explored the effects of an anthocyanin-rich bilberry extract in ulcerative colitis. Despite the fact that global microbial diversity was unchanged, the extract disrupted the pathological association between *Haemophilus parainfluenzae* and fecal calprotectin—a well-established clinical marker of intestinal inflammation. These results support the notion that dietary bioactives may modulate host–microbe interactions in ways that mitigate disease activity, even without overt changes in gut microbiome composition.

Additionally, within a pilot clinical study, Onyeaghala et al. (contributor 6) assessed how aspirin intervention modulates the oral microbiome, which is increasingly acknowledged as a contributor and common denominator to both gastrointestinal and systemic diseases, including colorectal carcinoma. Over six weeks, aspirin oral administration led to elevated relative abundances of *Neisseria*, *Streptococcus*, and *Actinomyces* while reducing taxa such as *Fusobacterium* and *Porphyromonas*, organisms enriched in IBD and colorectal cancer. Such preliminary results underscore the potential of common medications to reshape microbial balances in ways that may attenuate cancer risk—a promising concept that deserves, however, validation in further large-scale, metagenomic studies.

Moving from the oral cavity to the intestinal tract, Saha and Hartmann (contributor 11) reviewed the impact of microbiome alterations on gut permeability in chronic liver diseases and IBD. Their synthesis illustrates how deleterious microbial products may compromise the intestinal barrier, thereby fueling inflammation and disease progression. Therapeutic interventions targeting barrier function, including precision microbiome approaches, are discussed as promising strategies to alleviate chronic gastrointestinal and hepatobiliary conditions.

The interplay between the microbiome and systemic autoimmunity is exemplified by rheumatoid arthritis (RA). In a conceptual review, Taneja (contributor 12) underscores that while genetic factors, such as HLA alleles, provide strong susceptibility, the gut microbiome acts as a key environmental driver of disease onset and further progression. The review highlights certain microbial taxa and metabolites implicated in immune dysregulation, setting the microbiome as both a biomarker source and a potential treatment target in RA.

Furthermore, Alba et al. (contributor 5) profiled the fecal microbiota and immune signatures of children with Phelan–McDermid syndrome (PMS), a rare disease characterized by developmental delay and autism-spectrum-like behaviors. Compared to control counterparts, children with PMS revealed decreased abundances of beneficial taxa including *Faecalibacterium* and *Agathobacter*, alongside reduced SCFA concentrations. Immune profiling exhibited elevated pro-inflammatory cytokines, though not reaching statistical significance. The aforementioned findings propose that altered microbial composition and impaired metabolite production might contribute to the gastrointestinal and neurodevelopmental manifestations of PMS, pointing toward microbiota-targeted modulations as possible supportive therapies.

At the opposite end of the lifespan, Kapphan et al. (contributor 10) investigated how frailty and chronological age impact the microbiota in a murine model of Alzheimer’s disease (AD). They reported that frailty scores provided a more sensitive marker of microbiome alterations than age alone, with AD mouse model displaying decreased levels of *Bacteroides* species. This fact proposed that the “quality of aging”, as captured by frailty, might interact with disease processes to shape microbial communities. Incorporating frailty into microbiome research may further ameliorate our comprehension of neurodegenerative disease trajectories.

Animal models still remain indispensable for translating host–microbe interactions, offering mechanistic insights that cannot easily be obtained in humans. In this respect, Guo et al. (contributor 7) compared the gut bacterial and fungal populations of chinchillas, ferrets, and marmots, three substantial research models, used as substantial models for human infectious disease research. Their study reported striking interspecies differences, particularly in the mycobiome, with ferrets enriched in *Rhodotorula* and *Kurtzmaniella*, chinchillas in *Teunomyces* and *Penicillium*, and marmots in *Piromyces* and *Kernia*. In spite of shared bacterial taxa such as *Clostridium* and *Pseudomonas*, distinct microbial signatures were evident across species, reflecting both evolutionary divergence and dietary niches. These findings offer a valuable reference for interpreting translational evidence and highlight the necessity to account for host-specific microbial ecology when extrapolating from animal studies to human pathophysiology.

Moreover, Shaheen et al. (contributor 3) investigated the stability and efficacy of different FMT formulations for the management of recurrent *Clostridioides difficile* infection. Their study reported that although bacteria exhibited reduced viability during storage, clinical outcomes were not necessarily impaired, suggesting that microbial functional capacity might be more relevant than merely viability counts. Lyophilized preparations, in particular, displayed a preserved ability to generate SCFAs in vitro, highlighting therefore that therapeutic benefit may stem from maintaining essential microbial activities. The aforementioned findings add a critical translational perspective by underscoring that formulation and storage parameters can influence FMT performance, thereby signifying future optimization of microbiome-based treatments.

Within the cardiometabolic field, Caparrós-Martín et al. (contributor 9) investigated the effects of PCSK9 inhibition with alirocumab in statin-treated patients with increased lipoprotein (a). Although overall bacterial diversity and circulating SCFA profiles remained unchanged, therapy was linked to elevated fecal bile acid concentrations. This observation proposes a mechanistic association between cholesterol-lowering therapy and microbial bile acid metabolism, offering a novel paradigm on how cardiometabolic drugs may indirectly influence gut microbial functions.

This collection of articles, hosted as a Special Issue of *Microorganisms* MDPI, summarizes the extraordinary breadth of the gut microbiome’s impact across homeostasis and disease. Ranging from infections such as LNTB to chronic conditions (e.g., MASLD, IBD, and RA) and to rare genetic syndromes as well as neurodegenerative models, the thirteen compilations highlight that dysbiosis and disrupted microbial metabolism are unifying threads across diverse pathologies.

Several common patterns are identified: Firstly, the loss of SCFA-producing taxa and impaired microbial metabolite profiles characterizes a plethora of conditions ranging from tuberculosis to PMS and AD models, suggesting therefore a central role for microbial metabolic capacity in maintaining host resilience. Secondly, immune and barrier dysfunction constitute convergent mechanisms linking microbiome alterations to systemic inflammation and disease progression. Thirdly, interventions—whether through FMT, probiotics, or bioactive dietary compounds—display that microbiome modulation can alleviate disease, though often by refining host–microbiota interactions rather than inducing “wholesale” community restructuring. In this respect, recent reviews highlight that SCFA-producing bacteria, for instance, *Faecalibacterium* and *Roseburia*, exert striking anti-inflammatory effects, and their depletion might constitute a shared signature of chronic diseases. Moreover, next-generation therapies, including optimized FMT and microbial consortia, are emergingly being explored as strategies to restore metabolic and immune homeostasis [5,6].

These insights chart several proposals for future cutting-edge research. New mechanistic studies should move beyond descriptive correlation in order to establish causal pathways, integrating multi-omics approaches with functional validation in both preclinical models and carefully designed clinical trials. Greater attention should be paid to species- and strain-level differences, recognizing that genera such as *Blautia* harbor both protective and potentially pathogenic members. Moreover, translational research should account for host context, including genetic predisposition, frailty, and environmental exposures, all of which modulate microbiome–host interactions. Such challenges underscore the necessity for large, longitudinal, and multi-ethnic cohort studies that can validate etiologic linkage and ensure reproducibility across various populations [7].

Ultimately, the gut microbiota field stands at promising crossroads: Advancements in sequencing, metabolomics, and systems biology now enable the construction of integrative models that can guide precision medicine and microbiome interventions. Realizing this potential will demand collaborative endeavors, standardized methodologies, and data sharing in an interdisciplinary manner. In parallel, artificial intelligence along with machine learning approaches are increasingly applied to microbiome data, permitting integration of high-dimensional sequencing, metabolomic, and clinical variables into predictive models. Such “smart” tools are promising not only for identifying causal microbial signatures but also for stratifying patients and offering personalized therapeutic interventions. The convergence of artificial intelligence with multi-omics microbiome research may therefore accelerate the translation of complex datasets into clinically applicable insights [8]. As the contributions of the current Special Issue illustrate, the gut microbiome is not just a peripheral player but a cornerstone and key determinant of health and disease. Harnessing its complexity for therapeutic purposes still remains a challenge but also an unprecedented opportunity to transform medicine in the next decades.

## Abbreviations

AD	Alzheimer’s disease
AH	Arterial hypertension
FMT	Fecal microbiota transplantation
IBD	Inflammatory bowel disease
IgE	Immunoglobulin E
LNTB	Lymph node tuberculosis
MASLD	Metabolic (dysfunction)-associated steatotic liver disease
MetS	Metabolic syndrome
PMS	Phelan–McDermid syndrome
RA	Rheumatoid arthritis
SCFA(s)	Short-chain fatty acid(s)

## References

[1] Loh J.S., Mak W.Q., Tan L.K.S., Ng C.X., Chan H.H., Yeow S.H., Foo J.B., Ong Y.S., How C.W., Khaw K.Y. (2024). Microbiota-gut-brain axis and its therapeutic applications in neurodegenerative diseases. Signal Transduct. Target. Ther..

[2] Zheng D., Liwinski T., Elinav E. (2020). Interaction between microbiota and immunity in health and disease. Cell Res..

[3] Chetty A., Blekhman R. (2024). Multi-omic approaches for host-microbiome data integration. Gut Microbes.

[4] Basic M., Dardevet D., Abuja P.M., Bolsega S., Bornes S., Caesar R., Calabrese F.M., Collino M., De Angelis M., Gerard P. (2022). Approaches to discern if microbiome associations reflect causation in metabolic and immune disorders. Gut Microbes.

[5] Sahle Z., Engidaye G., Shenkute Gebreyes D., Adenew B., Abebe T.A. (2024). Fecal microbiota transplantation and next-generation therapies: A review on targeting dysbiosis in metabolic disorders and beyond. SAGE Open Med..

[6] Fusco W., Lorenzo M.B., Cintoni M., Porcari S., Rinninella E., Kaitsas F., Lener E., Mele M.C., Gasbarrini A., Collado M.C. (2023). Short-Chain Fatty-Acid-Producing Bacteria: Key Components of the Human Gut Microbiota. Nutrients.

[7] Lloyd-Price J., Arze C., Ananthakrishnan A.N., Schirmer M., Avila-Pacheco J., Poon T.W., Andrews E., Ajami N.J., Bonham K.S., Brislawn C.J. (2019). Multi-omics of the gut microbial ecosystem in inflammatory bowel diseases. Nature.

[8] Topol E.J. (2019). High-performance medicine: The convergence of human and artificial intelligence. Nat. Med..

